# Spontaneous Pneumomediastinum in a Patient With Severe COVID‐19 Pneumonia: A Case Report

**DOI:** 10.1002/ccr3.71042

**Published:** 2025-09-29

**Authors:** Merga Daba Mulisa, Abel Tenaw Tasamma, Sebhatleab Teju Mulate, Eskedar Ferdu Azerefegne

**Affiliations:** ^1^ Department of Internal Medicine, College of Health Sciences Addis Ababa University Addis Ababa Ethiopia; ^2^ Division of Infectious Diseases, Department of Internal Medicine, College of Health Sciences Addis Ababa University Addis Ababa Ethiopia

**Keywords:** complications, COVID‐19, Ethiopia, pneumomediastinum

## Abstract

Pneumomediastinum is a rare complication of Coronavirus disease 2019 (COVID‐19). It can occur spontaneously or as a complication of mechanical ventilation. Patients typically present with chest pain or shortness of breath. There are only a few case reports of pneumomediastinum complicating a course of COVID‐19 pneumonia. Here, we present a case of a 35‐year‐old male patient who presented with shortness of breath for 3 days. Subsequent evaluation using polymerase chain reaction (PCR) and a chest computed tomography (CT) scan revealed severe COVID‐19 pneumonia complicated by pneumomediastinum. He was managed conservatively for pneumomediastinum and was discharged with improvement.

**Summary**

- Pneumomediastinum is an uncommon complication associated with COVID‐19 pneumonia, and its diagnosis may be delayed or even overlooked, as observed in our patient.
- Hence, physicians should include pneumomediastinum in the differential diagnosis for chest pain in COVID‐19 patients, particularly when they do not respond to standard treatments for COVID‐19 pneumonia.

## 1 Introduction

Coronavirus disease 2019 (COVID‐19) is caused by severe acute respiratory syndrome coronavirus 2 (SARS‐CoV‐2) [1, 2, 3]. The clinical spectrum of this disease ranges from asymptomatic to severe or critical illness, with the most common symptoms being cough and fever. It has both acute and long‐term complications affecting all organ systems of the body. Common complications include acute respiratory distress syndrome, acute respiratory failure, sepsis, disseminated intravascular coagulation, acute liver and kidney injury, and pulmonary embolism [4, 5].

Spontaneous pneumomediastinum is an uncommon complication of COVID‐19. Here, we present a case of a 36‐year‐old male patient diagnosed with severe COVID‐19 pneumonia complicated by pneumomediastinum. We discuss this rare complication of the disease.

## 2 Case History and Physical Examination

A 36‐year‐old previously healthy male patient presented to our hospital with a complaint of shortness of breath for 4 days. He also experienced low‐grade fever, fatigue, vomiting, and watery diarrhea of the same duration. Further inquiry into his past medical history revealed that he had hypertension, for which he was being treated with amlodipine 10 mg orally daily.

Physical examination revealed an acutely ill‐looking man with a pulse rate of 120 beats per minute, a respiratory rate of 32 breaths per minute, blood pressure of 100/75 mmHg, and oxygen saturation of 75% on room air. Respiratory examination showed intercostal and subcostal retractions, fine crackles throughout the lung fields and bronchial breath sounds over the posterior chest.

A nasal sample was sent for severe acute respiratory syndrome coronavirus 2 (SARS‐CoV‐2) polymerase chain reaction (PCR), which returned positive. With the clinical impression of severe COVID‐19 pneumonia, our patient was treated with dexamethasone 6 mg intravenously twice‐daily, remdesivir for 4 days, cefepime 2 g intravenously three times‐daily, vancomycin 1 g intravenously twice‐daily, and morphine 2 mg intravenously four times daily. However, the patient experienced respiratory deterioration requiring admission to the intensive care unit (ICU) and continuous positive airway pressure (CPAP) therapy with the pressure of 6 mmHg, PEEP: 6 mmHg, FIO2: 60%. After 1 week in the ICU, the patient showed symptomatic improvement, except for hypoxemia, which complicated attempts to wean him off oxygen therapy.

## 3 Differential Diagnosis

At this point, the differential diagnosis considered for the patient's persistent desaturation and difficult weaning off mechanical ventilation was neuromuscular weakness, excessive sedation, electrolyte abnormalities, tension pneumothorax, and pulmonary thromboembolism (PTE).

## 4 Outcome and Follow‐Up

**FIGURE 1:**
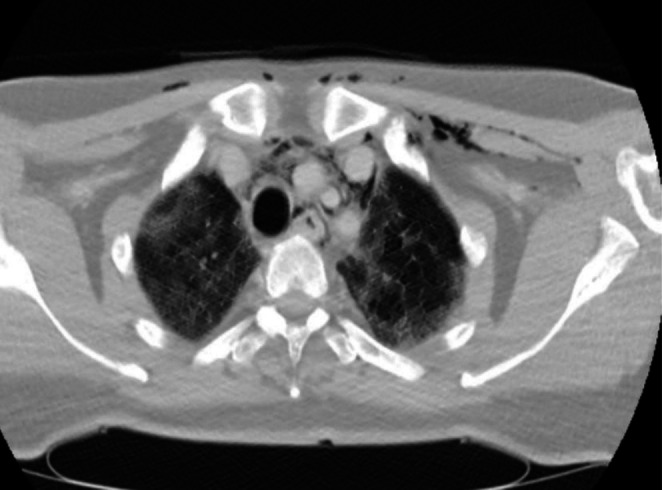
Non‐contrast CT scan of the chest: A Single representative slice image of a non‐contrast chest CT scan on Lung window at the level of T3 (above the arch of aorta) demonstrates bilateral diffuse mixed consolidation and GGO with interlobular smooth septal thickenings (crazy paving appearance). It also shows free air in the mediastinum surrounding the major vessels and the airway (pneumomediastinum) as well subcutaneous emphysema over anterior chest wall and lower neck more exuberant on the left side.

**FIGURE 2:**
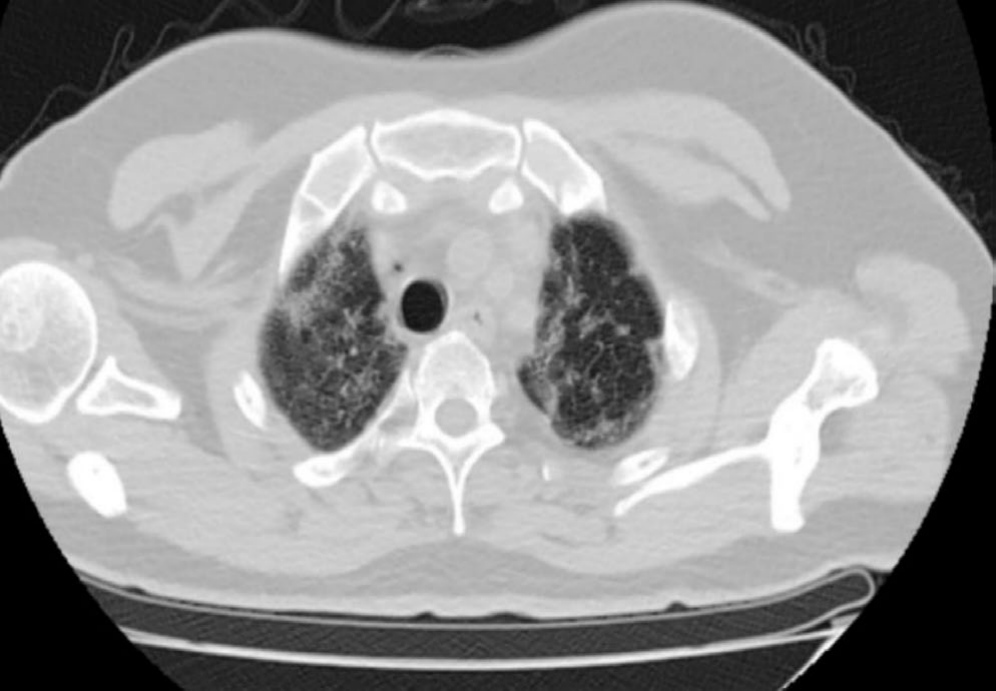
Follow‐up non‐contrast chest CT on lung window at the level just above the arch of the aorta shows bilateral patchy consolidation with interlobular septal thickening with reticulation and architectural distortion demonstrating early post‐COVID‐19 fibrotic changes. It also shows resolution of the pneumomediastinum and subcutaneous emphysema.

A subsequent chest computed tomography (CT) scan revealed free air in the mediastinum, confirming the diagnosis of pneumomediastinum (Figure 1). High‐flow oxygen therapy was initiated, and after 3 days, the patient showed clinical improvement, allowing for the discontinuation of oxygen therapy. A follow‐up chest computed tomography (CT) scan showed resolution of the pneumomediastinum (Figure 2), and he was discharged with improvement.

## 5 Discussion

Pneumomediastinum, the presence of air in the mediastinum, is a rare complication of COVID‐19 pneumonia. The UK POETIC survey reported an incidence of spontaneous primary pneumomediastinum in COVID‐19 patients of 0.13%. Although it may occur as a direct consequence of COVID‐19, airway trauma during tracheal intubation, barotrauma, or repositioning maneuvers can also contribute [6, 7, 8, 9]. COVID‐19 pneumonia may produce cystic lung lesions that can either disappear or develop into larger blebs. Rupture of these cysts may lead to spontaneous pneumothorax or pneumomediastinum and subcutaneous emphysema [7, 10, 11]. Pneumomediastinum is classified as primary (spontaneous) and secondary, with the latter further divided into iatrogenic, traumatic, and nontraumatic. Most pneumomediastinum cases in COVID‐19 patients are attributed to positive pressure ventilation [12].

In patients with COVID‐19 pneumonia, pneumothorax and subcutaneous emphysema have been reported concurrently with pneumomediastinum in about 40% and 75% of cases, respectively [13]. Secondary spontaneous pneumothorax has been reported as a consequence of COVID‐19 in 1% of hospitalized patients [14], 3% in hospitalized patients with pneumonia [15], and 6% in mechanically ventilated patients [16]. Pneumothorax can occur in critically ill COVID‐19 pneumonia patients who are breathing spontaneously or mechanically ventilated and can be unilateral or bilateral [17].

Our patient had severe COVID‐19 pneumonia complicated by both pneumomediastinum and subcutaneous emphysema. He was treated with continuous positive pressure ventilation and did not require intubation or mechanical ventilation. After 3 days, he was weaned off oxygen therapy and discharged with improvement. Follow‐up chest computed tomography (CT) confirmed the resolution of the pneumomediastinum. There are case reports, including our patient, that describe the late diagnosis of pneumothorax and pneumomediastinum [18, 19, 20]. This underscores the importance of maintaining a high index of suspicion for pneumomediastinum and performing early imaging in patients who do not respond to initial therapy.

## 6 Conclusion

Pneumomediastinum is a rare complication of COVID‐19 pneumonia. Its diagnosis can be delayed, as in our patient, or missed altogether. Therefore, physicians should consider pneumomediastinum in the differential diagnosis of chest pain in COVID‐19 patients, especially if they do not respond to standard treatments for COVID‐19 pneumonia.

## Author Contributions

**Merga Daba Mulisa:** conceptualization, writing – original draft. **Abel Tenaw Tasamma:** conceptualization, writing – review and editing. **Sebhatleab Teju Mulate:** writing – original draft. **Eskedar Ferdu Azerefegne:** conceptualization, writing – review and editing.

## Ethics Statement

The author's institution does not require ethics approval for the publication of a single case report.

## Consent

Written informed consent was obtained from the patient for publication and any accompanying images.

## Conflicts of Interest

The authors declare no conflicts of interest.

## Data Availability

Data sharing not applicable to this article as no datasets were generated or analyzed during the current study.
